# Oxytocin treatment attenuates amygdala activity in autism: a treatment-mechanism study with long-term follow-up

**DOI:** 10.1038/s41398-020-01069-w

**Published:** 2020-11-06

**Authors:** Sylvie Bernaerts, Bart Boets, Jean Steyaert, Nicole Wenderoth, Kaat Alaerts

**Affiliations:** 1 Department of Rehabilitation Sciences, Research Group for Neurorehabilitation, KU Leuven, Leuven, Belgium; 2 Leuven Autism Research (LAuRes), KU Leuven, Leuven, Belgium; 3 Department of Neurosciences, Center for Developmental Psychiatry, KU Leuven, Leuven, Belgium; 4 Department of Health Sciences and Technology, Neural Control of Movement Lab, ETH Zurich, Zurich, Switzerland

**Keywords:** Autism spectrum disorders, Neuroscience

## Abstract

Intranasal administration of the neuropeptide oxytocin (IN-OT) is increasingly considered as a potential treatment for targeting the core symptoms of autism spectrum disorder (ASD), but the effects of continual use on neural substrates are fairly unexplored and long-term effects are unknown. In this double-blind, randomized, placebo-controlled study, we investigated the effects of single-dose and multiple-dose IN-OT treatment (4 weeks of daily (24 IU) administrations) on brain activity related to processing emotional states. Thirty-eight adult men with ASD (aged between 18 and 35 years) underwent functional magnetic resonance imaging of the posterior superior temporal gyrus (pSTS) and amygdala regions while processing emotional states from point-light biological motion. In line with prior research, a single dose of IN-OT induced a reliable increase in pSTS brain activity during the processing of point-light biological motion, but no consistent long-term changes in pSTS activity were induced after the multiple-dose treatment. In terms of bilateral amygdala, the multiple-dose treatment induced a consistent attenuation in brain activity, which outlasted the period of actual administrations until four weeks and one year post-treatment. Critically, participants with stronger attenuations in amygdala-activity showed greater behavioral improvements, particularly in terms of self-reported feelings of avoidant attachment and social functioning. Together, these observations provide initial insights into the long-lasting neural consequences of chronic IN-OT use on amygdala functioning and provide first indications that the acute versus chronic effects of IN-OT administration may be qualitatively different. Larger studies are however warranted to further elucidate the long-term impact of IN-OT treatment on human neural substrates and its behavioral consequences.

## Introduction

Intranasal administration of the neuropeptide oxytocin (IN-OT) is increasingly explored as a potential pharmacotherapy for targeting the core symptoms of autism spectrum disorder (ASD), a prevalent neurodevelopmental disorder characterized by impairments in social and communicative functioning and stereotyped and repetitive behavior. OT is a neuropeptide produced by the paraventricular and supraoptic nuclei of the hypothalamus and has been implicated to act as an important neuromodulator for mediating a wide range of complex social behaviors, including interpersonal bonding, attachment, and affiliative and cooperative behavior (reviewed in refs. ^1–3^).

To date, initial single-dose administration studies in ASD have consistently demonstrated behavioral improvements on various social tasks^4–8^ (reviewed in ref. ^9^). Also several multiple-dose administration studies showed clinical improvements in ASD symptoms after 4, 5, or 6 weeks of multiple-dose IN-OT administration in adults^10–13^ and young children with ASD^14,15^, even though other multiple-dose studies failed to replicate positive clinical outcomes after 4-day or 8-week trials in children^16^ and adolescents with ASD^17^.

Mechanistically, OT’s impact on human brain function has been proposed to involve a bottom-up anxiolytic effect to facilitate social approach behavior, and a top-down social salience effect to facilitate attention to, and perception of social signals^18–21^. In line with the top-down social salience effect, initial neuroimaging studies (both in typically developing individuals (reviewed in ref. ^22^) and individuals with ASD^5,23–28^ provided evidence that single-dose IN-OT administration can alter activations in brain regions involved in socio-communicative processing and salience networks, by modulating attention to and perception of biologically relevant stimuli, such as social information conveyed by eyes, faces, or biological motion^20^. For example, previous IN-OT administration studies have demonstrated improved biological motion perception^29–31^ and increased activity in the posterior superior temporal sulcus (pSTS)—a core brain region involved in socio-communicative processing^23^.

IN-OT studies have also consistently highlighted the involvement of the amygdala^32^, a key neural structure associated with the detection and processing of emotionally relevant stimuli and modulation of emotional arousal (e.g. ref. ^33^). From animal research, OT has been implicated in reducing amygdala reactivity through inhibitory GABAergic interneurons^34^, a notion that is generally supported by previous human task-based fMRI studies showing attenuated amygdala responses after single-dose IN-OT administration^35–46^ (in ASD^26,28^). However, a number of studies have also reported increased amygdala activation following IN-OT administration^47–50^ (in ASD^5,23,25^), and several person-dependent (e.g., sex, psychopathology), and/or contextual factors (e.g., task type, stimulus valence, dose) have been put forward as potential sources of variability (reviewed in refs. ^32,51^). Particularly, since the amygdala forms an integral part of the threat-processing circuit, as well as the (social) salience network, it has been suggested that opposite directions of IN-OT effects on amygdala activity may reflect its multifaceted action at the level of the brain^20^. Specifically, as proposed by the influential ‘Social adaptation model’ of OT, increased amygdala reactivity following IN-OT administration is suggested to reflect a neural mechanism for facilitating attention to, and processing of salient (social) cues. Attenuation of amygdala reactivity, on the other hand, is anticipated to reflect OT’s anxiolytic role in down-regulating negative affect, social withdrawal and distress^20^. Accordingly, the direction of IN-OT’s effect on neural substrates (reducing or facilitating activation) is suggested to depend primarily on the required (social) adaptation^20^. For example, as outlined in Ma et al. (2016)^20^, for individuals with excessive social fear and stress (for example, in individuals with social anxiety disorder), IN-OT may primarily attenuate excessive amygdala reactivity^41^, whereas for individuals with low baseline social sensitivity (for example, in individuals with ASD), IN-OT may primarily enhance social salience and the neural substrates underlying social processing (e.g. IN-OT induced increases in amygdala activity in ASD^5,23,25^, but see refs. ^26,28^).

To date, insights into the neural effects of IN-OT have mostly emerged from single-dose administration studies. One prior study evaluated the neural effects of multiple-dose IN-OT treatment in adults with ASD and demonstrated reliable changes in functional connectivity and task-related brain activity in anterior cingulate and prefrontal regions during a social-judgment task after 6 weeks of IN-OT treatment. However, due to the use of a cross-over design, this latter study did not include follow-up assessments to evaluate possible long-lasting neural effects that outlast the period of actual IN-OT administration. These evaluations are important, however, since the possibility cannot be ruled out that repeated use of IN-OT over a longer period of time may induce long-lasting changes in circuits of the social brain and/or central oxytocinergic system. Indeed, in light of the growing number of trials assessing clinical responses of multiple-dose IN-OT treatment in ASD^10,11,14–17^, it seems crucial to gain a deeper mechanistic understanding into the neural substrates that underlie behavioral effects of multiple-dose IN-OT treatment, and, in particular, to evaluate the possibility of long-lasting neural consequences after chronic OT use. These insights will be essential for endorsing a further translation and development of OT-based therapies to clinical settings.

In the current study with randomized, double blind, placebo-controlled parallel design, we aimed to examine the (long-lasting) effects of IN-OT treatment on the neural substrates underlying socio-communicative processing in adult men with ASD. Neural functioning was assessed before and after IN-OT treatment using a functional magnetic resonance imaging (fMRI) paradigm involving the processing of emotional states from biological motion conveyed by whole-body point-light displays (PLDs). Importantly, the current study did not only assess the immediate, neural effects induced after administration of a *single dose* of IN-OT. Also the neural effects of *multiple-dose* treatment (4 weeks of once daily administrations, 24 IU/day) and the possibility of long-lasting neural *retention* effects were assessed in the same sample of individuals with ASD. Specifically, fMRI scanning was performed: (i) at baseline; (ii) 30 min after the single-dose administration; (iii) after the 4-week multiple-dose treatment, and at two follow-up sessions; (iv) 4 weeks; and (v) one year post-treatment.

In line with the social adaptation model of OT^20^ and prior IN-OT administration studies^23^, we primarily hypothesized a single dose of IN-OT to *enhance* neural recruitment of pSTS regions, a key neural structure involved in point-light biological motion perception and highlighted as an area of dysfunction in ASD (reviewed in ref. ^52^). Similarly, we also primarily hypothesized a single dose of IN-OT to *enhance* amygdala activity upon processing of emotional states from biological motion; i.e., being reflective of an increased social sensitivity towards the presented point-light stimuli after IN-OT^20^. However, considering the complex role of the amygdala and inconclusive results from prior single-dose administration studies in ASD (reporting either increases^5,23,25^ of decreases^26,28^ in amygdala activity after IN-OT), the possibility of finding an attenuating effect of IN-OT on amygdala reactivity was not ruled out (i.e., reflecting OT’s anxiolytic role in downregulating negative affect, social withdrawal and distress).

Crucially, a key aim was to determine whether the multiple-dose treatment could induce similar or even augmented changes in pSTS and amygdala activity, and specifically, whether the changes in neural function would outlast the period of actual administration until 4 weeks or even one year post-treatment. Previous analyses within the same sample of individuals with ASD^13^ have revealed that the 4-week IN-OT treatment improved symptoms of autism, most notably in terms of repetitive behaviors (as assessed with the Repetitive Behavior Scale-Revised: RBS-R); feelings of attachment avoidance (as assessed with the State Adult Attachment Measure: SAAM) and, to a more variable extent, social functioning (as assessed with the Social Responsiveness Scale for Adults: SRS-A). To shed more light on the neural basis of these clinical responses, a key aim of the current study was to explore ‘brain–behavior’ relationships, i.e., assessing whether OT-induced neural changes are related to these prior identifications of long-lasting behavioral improvements in terms of repetitive behaviors and attachment and/or social functioning (see “Materials and methods” and ref. ^13^).

## Materials and methods

### General study design

**Fig. 1:**
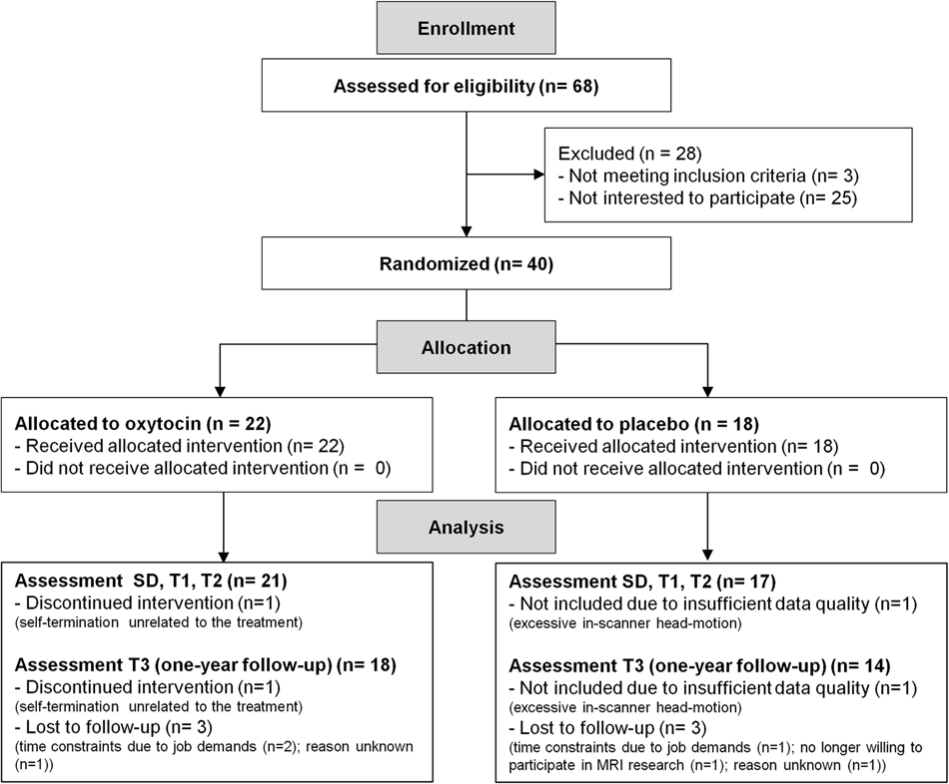
CONSORT flow diagram. MRI scanning was performed at baseline (T0); after a single dose of oxytocin or placebo treatment (SD); after the 4-week (oxytocin/placebo) treatment (T1); and at two follow-up sessions, 4 weeks (T2) and one year after cessation of the treatment (T3). One participant of the oxytocin group was excluded from the analyses, due to self-termination of participation (unrelated to the treatment). One participant of the placebo group was excluded from the MRI analyses due to excessive in-scanner head motion (mean frame-wise displacement exceeding > 0.5 mm). Six additional participants (three oxytocin, three placebo) were lost for follow-up session T3.

In this two-arm, double-blind, randomized, placebo-controlled parallel study, we assessed single-dose and multiple-dose effects of intranasal oxytocin (IN-OT) administration on brain activity during emotion processing from point-light biological motion in adult men with ASD. Changes-from-baseline (T0) in brain activity were assessed immediately after a single dose of IN-OT treatment (SD), after 4 weeks of daily IN-OT administrations (T1), and at two follow-up sessions, 4 weeks (T2) and one year post-treatment (T3) (Fig. 1, CONSORT flow diagram for number of participants included in each assessment session). The neural assessments were conducted at the Leuven University Hospital (Leuven, Belgium) in the context of a larger study, also including behavioral assessments (reported in ref. ^13^) and other scan modalities (e.g. resting-state fMRI, reported in ref. ^53^) (registered at the EU Clinical Trials register: Eudract 2014-000586-45 and clinicaltrial.gov: NCT02940574). Written informed consent was obtained from all participants prior to the study. Consent forms and study design were approved by the local Ethics Committee for Biomedical Research at the University of Leuven, KU Leuven (S56327) in accordance to The Code of Ethics of the World Medical Association (Declaration of Helsinki).

### Participants

Participants were mainly recruited from the Autism Expertise Centre at the Leuven University Hospital between April 2015 and December 2016. ASD diagnosis was made by a multidisciplinary team based on the strict criteria of the DSM-IV-TR^54^. Prior to the treatment, the Autism Diagnostic Observation Schedule (ADOS)^55^ and estimates of intelligence (six-subtest short-version of the Dutch Wechsler Adult Intelligence Scale-IV)^56^ were acquired from all participants (Table 1).

**Table 1:** Participant characteristics.

	Oxytocin	Placebo
Number of participants	21	17
Age	24.76 ± 4.85	24.06 ± 5.54
Handedness	5L/16R	2L/15R
*IQ*		
Total IQ	102 ± 13	107 ± 19
VIQ	106 ± 9	111 ± 13
PIQ	105 ± 18	104 ± 22
*ADOS-2*		
Total	7.2 ± 4.3	7.6 ± 3.9
Communication	2.1 ± 1.4	2.2 ± 1.4
Social interaction	5.1 ± 3.4	5.4 ± 3.1
Use of psychostimulant medication	5	2
Comorbidity	7	2

Data are shown as mean ± standard deviation. Detailed information on medication use and comorbidities is provided in Supplementary Table 1.

*L* left-handed, *R* right-handed, *IQ* intelligence quotient, *VIQ* verbal IQ, *PIQ* performance IQ, *ADOS* autism diagnostic observation schedule.

Inclusion criteria comprised clinical diagnosis of ASD; gender (male); and age (18–35 years old). Exclusion criteria for participation comprised any neurological disorder (e.g., stroke, epilepsy, concussion); demonstrated genetic disorder; or any contraindication for MRI. Current psychoactive medication use and the presence of comorbid psychiatric disorders were screened (Table 1, Supplementary Table 1). As described in more detail in Supplementary Methods, sample sizes were based on a prior fMRI study assessing the neural effects of multiple-dose IN-OT treatment in adults with ASD^11^.

### Intervention

Participants were assigned to receive IN-OT or PL based on a computer-generated randomized order. Except for the manager of randomization, all research staff conducting the trial, participants and their parents and/or partners were blinded to treatment allocation. IN-OT (Syntocinon®, Sigma-tau) and PL (saline sodium-chloride solution) were administered in amber 15 ml glass bottles with metered pump (ACA Pharma). Each puff per nostril contained 4 international units (IU) of OT.

Participants self-administered a daily dose of 24 IU (3 puffs/nostril) over 4 consecutive weeks (28 doses in total). This dose is in accordance with prior studies assessing single-dose effects of IN-OT on social cognition (for review see ref. ^3^). All participants administered the first dose in front of the experimenter and received clear instructions about the use of the nasal spray in accordance with recommendations by Guastella et al. (2013)^57^. At first use, air present in the nasal spray was removed by pumping the spray until a fine mist was observed. Participants were instructed to keep one nostril closed, to take a deep breath through the nose and to tilt their head slightly backwards during nasal administration in order to minimize gravitational loss of the spray. During the course of the treatment, participants were asked to administer the nasal spray in the morning, to keep a daily record of the time point of nasal spray administration, and whether or not they were alone or in company of others the first two hours after administration. Percentage of days at which the spray was administered in the presence of others was similar for the OT (36.6% SD 29.9) and PL (37.4% SD 24.1) treatment group.

All participants were monitored onsite until approximately two hours after first nasal spray administration. More detailed information on side effect screening is provided in Supplementary Methods and Bernaerts et al. (2020)^13^.

### MRI data acquisition and handling

#### MRI data acquisition

Anatomical and task-related fMRI images were acquired on a 3.0 Tesla Philips MR scanner (Best, The Netherlands) with an 8-channel phased-array head coil at baseline (T0); after a single dose of IN-OT (~45 min after administration) (SD); after 4 weeks of continual IN-OT treatment (at least 24 h after the final administration) (T1); and at two follow-up sessions, 4 weeks (T2) and 1 year post-treatment (T3). Detailed information on the scanning parameters is provided in Supplementary Methods.

#### fMRI experimental paradigm

During task-based functional MRI scanning, participants performed an emotion processing task (based on prior work from our lab^31,58–60^, involving the recognition of positive and negative emotional states (happiness, anger) from whole-body PLDs (see Supplementary Fig. 1A for a visualization of an exemplary PLD) (see ref. ^61^ for a detailed description of the adopted PLDs). During fMRI scanning, participants performed six blocks of the emotion task (12 trials/block, 4 s each) interleaved with six blocks of a control task, involving detection of color changes in scrambled versions of the PLDs. Rest blocks (12 s) were presented between task blocks (fixation on white cross) (see Supplementary Fig. 1A). During each trial of the emotion task, participants were instructed to indicate as fast and accurately as possible whether the presented PLD figure conveyed happiness or anger by pressing the respective response buttons (happy or angry) (Supplementary Fig. 1B). During the control task, participants were presented with a scrambled version of the PLDs in which one dot changed color to red or green at a random time point. Participants were again instructed to indicate as fast and accurate as possible the color of the dot by pressing the respective response buttons (green or red). No feedback was provided after the responses. Task instructions were provided verbally and on the monitor at the start of each block. Prior to scanning, participants practiced the tasks (12 trials/task). Reaction times and accuracy rates were recorded using E-Prime-software (Psychological Software Tools). During each assessment session (T0, SD, T1, T2, T3), the same set of stimuli were presented in a randomized order.

#### fMRI data analysis

SPM8 (Wellcome Department of Imaging Neuroscience, University College London, UK) was used for image preprocessing and statistical analyses implemented in Matlab R2017b (Mathworks). Detailed information on preprocessing and head motion analysis is provided in Supplementary Methods and Supplementary Fig. 2. For each subject, a first-level general linear model was calculated with the time course of emotion and control blocks modeled as predictors and realignment parameters and task instructions as regressors of no interest. Separate epoch regressors were created for trials presenting happiness and anger. Contrast images were calculated for emotion (happiness) > control and emotion (anger) > control and were subjected to second-level random-effects models. First-level contrast images were then used to extract contrast estimates (MarsBar) from predefined regions of interest (ROIs) centered over bilateral pSTS (10-mm-radius spheres with MNI-coordinates [left: −55, −52, 12] [right: 55, −52, 10] based on ref. ^60^) and bilateral amygdala (structurally defined based on the FSL Harvard-Oxford subcortical atlas). Whole-brain one-sample *t*-test analysis identifying regions with brain activity during the emotion task (>control task) at the baseline session (T0) (across groups) confirmed that predefined ROIs (bilateral pSTS and amygdala) were reliably activated during the emotion task (*p* < 0.05, family-wise error (FWE) corrected for multiple comparisons) (Supplementary Fig. 3).

### Statistical analyses

#### Task-related fMRI

To explore treatment-induced changes in task-related brain activity, changes from baseline (T0) in average ROI activation levels (raw contrast estimates) were calculated (separately for each assessment session: SD, T1, T2, T3) and subjected to mixed-effects analyses with ‘Subject’ as random factor and the factors ‘Treatment’ (IN-OT, PL), ‘Emotion’ (Happiness, Anger) and ‘Hemisphere’ (Left, Right) as fixed factors. Separate models were constructed to explore treatment-induced pre-to-post changes in pSTS and amygdala activity after a single-dose administration (SD) or after multiple-dose treatment (T1, T2, T3).

#### Behavioral task performance

Performance accuracy (percentage of correct answers) and correct reaction times on the emotion task were assessed for each participant and assessment session, and a performance variable (accuracy divided by reaction time) was calculated. Note that across participants, a few trials of the emotion task were lost due to ‘no recorded response’ (T0: 3.58%, SD: 1.17%, T1: 2.12%, T2: 0.95%, T3: 1.87%). Similar to the neural analyses, mixed-effects analyses were performed to assess treatment-induced pre-to-post changes in behavioral performance. The significance level was set at *p* < 0.05. Post-hoc analyses were Bonferroni-corrected for multiple comparisons.

#### Association with behavioral improvements

As outlined in more detail in Bernaerts et al. (2020)^13^, we previously reported treatment-specific improvements until 4 weeks and even 1 year post-treatment in repetitive behaviors (assessed with the Repetitive Behavior Scale—Revised (RBS)^62^) and feelings of attachment avoidance (assessed with the SAAM^63^) induced by the multiple-dose IN-OT treatment (assessed within the same participant sample). Also, improvements in social functioning (assessed with the Social Responsiveness Scale (SRS)^64^) were identified, but these improvements were evident in both the treatment and the placebo group (see Supplementary Table 2 and Bernaerts et al. (2020)^13^ for a detailed description of treatment-induced changes in these behavioral measures).

Here, exploratory Pearson’s *r* correlation analyses were performed to assess whether the identified neural changes in brain activity were related to these previously identified behavioral changes. Considering the exploratory nature of these brain–behavior analyses, results are reported at an uncorrected *p* < 0.05 threshold. All statistics were performed with Statistica 8 (StatSoft. Inc., Tulsa, USA).

## Results

### Effect of IN-OT treatment on pSTS activity

#### Single-dose IN-OT administration

**Fig. 2:**
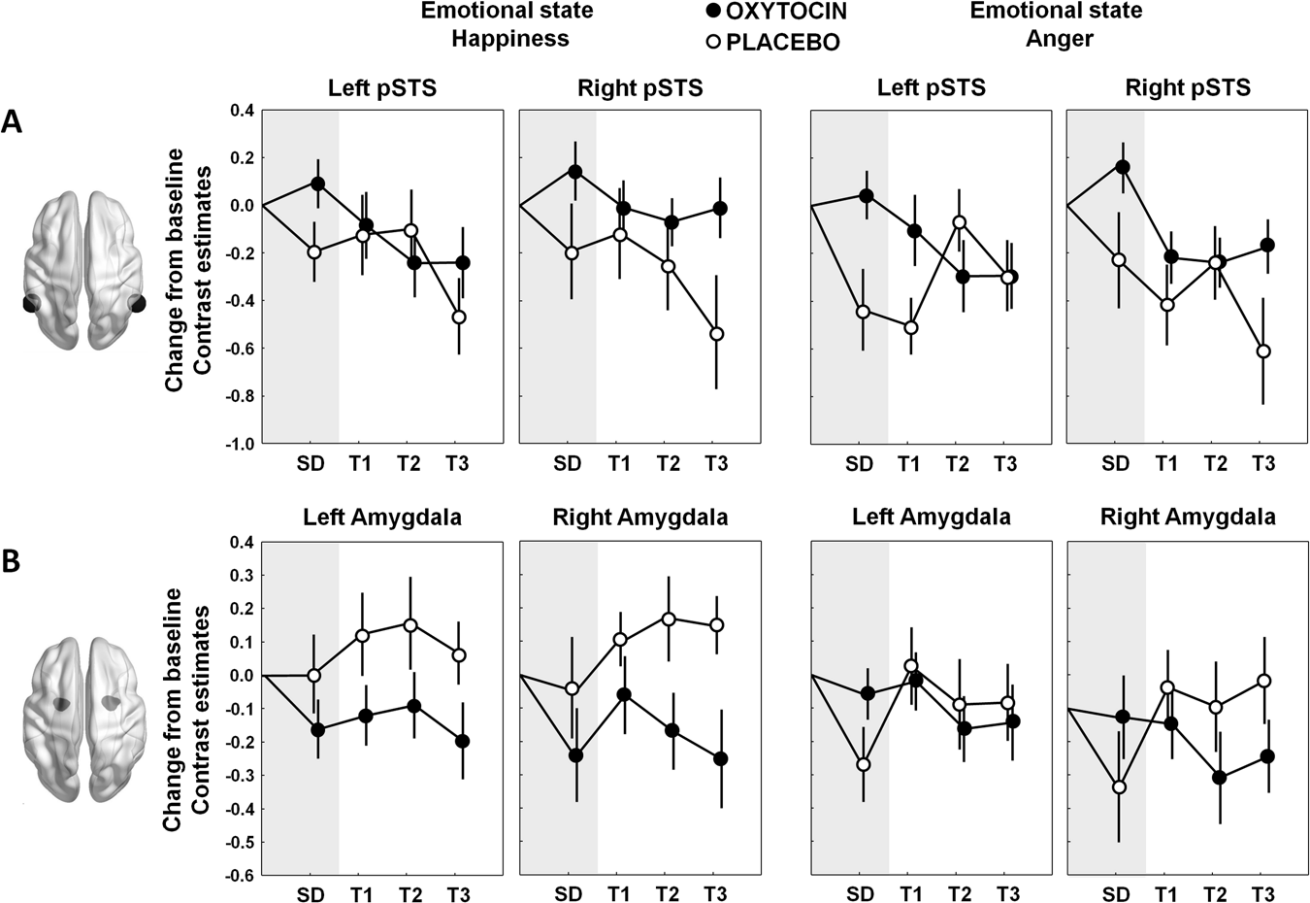
Effect of oxytocin treatment on task-related brain activity. Mean changes from baseline in neural activations during the processing of emotional states from point-light biological motion of the posterior superior temporal sulcus (pSTS) (panel **A**) and amygdala (panel **B**) are visualized separately for the oxytocin (IN-OT) and placebo (PL) treatment groups. Changes in neural activations are visualized separately for each emotional state (happiness, anger), hemisphere (left, right) and assessment session: SD (after a single-dose administration), T1 (immediately after the 4-week, multiple-dose treatment), T2 (at follow-up, 4 weeks post-treatment) and T3 (at follow-up, one year post-treatment). Bilateral pSTS and amygdala regions are visualized on inflated surface maps generated using BrainNet Viewer. Vertical bars denote ± standard errors.

Mixed-effects analyses with the fixed factors ‘treatment’ (IN-OT, PL), ‘emotion’ (happiness, anger), and ‘hemisphere’ (left, right) revealed a main effect of treatment (*F*(1, 36) = 5.33, *p* = 0.027, *d* = 0.74), indicating a significant stronger pre-to-post increase in brain activity of the pSTS during emotion processing from point-light biological motion after a single-dose of IN-OT, compared to PL. No significant interactions between treatment and the factors hemisphere (*F*(1, 110) = 0.03, *p* = 0.87) or emotion (*F*(1, 110) = 0.81, *p* = 0.37) were revealed, indicating that the main effect of treatment was evident irrespective of pSTS region (left, right) or processed emotional state (happiness, anger) (see Fig. 2A).

#### Multiple-dose IN-OT administration

Mixed-effects analyses with the fixed factors ‘treatment’ (IN-OT, PL), ‘emotion’ (happiness, anger), ‘hemisphere’ (left, right), and ‘session’ (T1, T2, T3) revealed a treatment-by-session interaction (*F*(2, 386) = 3.51, *p* = 0.031), indicating a significant effect of the multiple-dose treatment on pSTS activity only at the one-year follow-up session (T3: *p*_Bonferroni_ = 0.032, *d* = 0.62), but not immediately after the multiple-dose treatment (T1: *p*_Bonferroni_ = 0.49, *d* = 0.39) nor at the follow-up session, 4 weeks post-treatment (T2: *p*_Bonferroni_ = 1.00, *d* = −0.10). As seen in Fig. 2A, pSTS activity attenuated in both treatment arms, but at the one-year follow-up session, the attenuation was more pronounced in the PL compared to the OT group. Note that, at trend level, also a treatment-by-hemisphere interaction was revealed (*F*(1, 386) = 3.10, *p* = 0.079), indicating that the treatment effect (across sessions T1–T3) was significant for right pSTS (OT versus PL: *p*_Bonferroni_ = 0.014), but not for left pSTS (*p*_Bonferroni_ = 1.00) (Fig. 2A). The interaction between treatment and emotion was not significant (*F*(1, 386) = 0.01, *p* = 0.90).

### Effect of IN-OT treatment on amygdala activity

#### Single-dose IN-OT administration

Mixed-effects analyses with the fixed factors ‘treatment’ (IN-OT, PL), ‘emotion’ (happiness, anger), and ‘hemisphere’ (left, right) revealed no significant main effect of ‘treatment’ (*F*(1, 36) = 0.01, *p* = 0.92, *d* = 0.03), indicating that amygdala activity during PLD emotion processing was not significantly altered after a single dose of IN-OT, compared to PL (Fig. 2B). However, a significant treatment-by-emotion interaction was observed (*F*(1, 110) = 10.11, *p* = 0.002), indicating that in the OT group, amygdala attenuation was evident irrespective of emotional state (happiness versus anger: *p*_Bonferroni_ = 1.00), while in the PL group, amygdala attenuation was more pronounced upon presentation of point-light biological motion conveying a negative (anger) compared to a positive (happiness) emotional state (*p*_Bonferroni_ = 0.013) (Fig. 2B). The interaction between treatment and hemisphere was not significant (*F*(1, 110) = 0.03, *p* = 0.87).

#### Multiple-dose IN-OT administration

Mixed-effects analyses with the fixed factors ‘treatment’ (IN-OT, PL), ‘emotion’ (happiness, anger), ‘hemisphere’ (left, right), and ‘session’ (T1–T3) revealed a main effect of treatment (*F*(1, 386) = 4.41, *p* = 0.043), indicating that the multiple-dose IN-OT treatment induced an attenuation in amygdala activity across assessment sessions T1–T3 (no significant treatment-by-session interaction was evident: *F*(2, 386) = 0.49, *p* = 0.61) (Fig. 2B). The interaction between treatment and hemisphere was not significant (*F*(1, 386) = 1.09, *p* = 0.30), indicating that the treatment effect was evident irrespective of amygdala region (left, right).

Similar to the single-dose analysis, a trend-level treatment-by-emotion interaction was revealed (*F*(1, 386) = 3.65, *p* = 0.057), indicating that in the IN-OT group, amygdala attenuation was evident irrespective of emotional state (happiness versus anger: *p*_Bonferroni_ = 1.00), whereas in the PL group, amygdala attenuation was more pronounced upon presentation of point-light biological motion conveying the negative (anger) compared to the positive (happiness) emotional state (*p*_Bonferroni_ = 0.018) (Fig. 2B).

### Effect of IN-OT treatment on emotion recognition from PLDs

#### Single-dose IN-OT administration

**Fig. 3:**
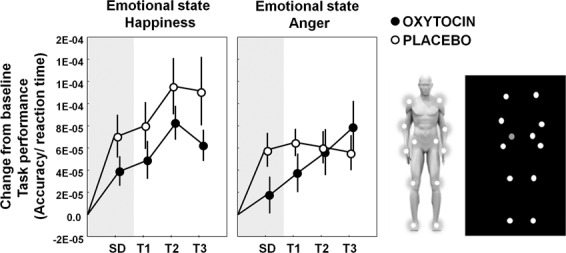
Effect of oxytocin treatment on emotion recognition from point-light displays. Mean changes from baseline in task performance (accuracy/reaction time) on the emotion task are visualized separately for the oxytocin (IN-OT) and placebo (PL) treatment groups. Changes in task-performance are visualized separately for each emotional state (happiness, anger) and assessment session: SD (after a single-dose administration), T1 (immediately after the 4-week, multiple-dose treatment), T2 (at follow-up, 4 weeks post-treatment), and T3 (at follow-up, one year post-treatment). Vertical bars denote ±standard errors. On the right, an exemplary point-light display is visualized, consisting of 12 white dots moving against a black background, representing the motion of the main joints of the human body (ankles, knees, hips, wrists, elbows, and shoulders).

Mixed-effects analyses with the fixed factors ‘treatment’ (IN-OT, PL) and ‘emotion’ (happiness, anger) revealed a main effect of treatment (*F*(1, 36) = 5.49, *p* = 0.025), which, contrary to our expectations, indicated that emotion recognition performance was improved in the PL group, compared to IN-OT group. No treatment-by-emotion interaction was evident (*F*(1, 36) = 0.075, *p* = 0.79), indicating that the performance gain of the PL group was evident irrespective of emotional state (Fig. 3).

#### Multiple-dose OT administration

Mixed-effect analysis with the fixed factors ‘treatment’ (OT, PL), ‘emotion’ (happiness, anger), and ‘session’ (T1–T3) assessing the effect of multiple-dose IN-OT treatment on task performance revealed no significant main effect of treatment (*F*(1,35.5) = 2.08, *p* = 0.13), nor a significant interaction of treatment with the factor ‘session’ (*F*(1,168) = 0.24, *p* = 0.79) or emotion (*F*(1,168) = 2.86, *p* = 0.092) (Fig. 3).

### Exploration of brain–behavior associations

#### Single-dose IN-OT administration

No significant association was identified between the observed increase in pSTS activity after the single-dose IN-OT administration (SD) and changes in task performance on the point-light emotion recognition task (Pearson correlation: OT: *r*_(21)_ = −0.30; *p* = 0.19; PL: *r*_(17)_ = 0.06; *p* = 0.81).

#### Multiple-dose IN-OT administration

**Fig. 4:**
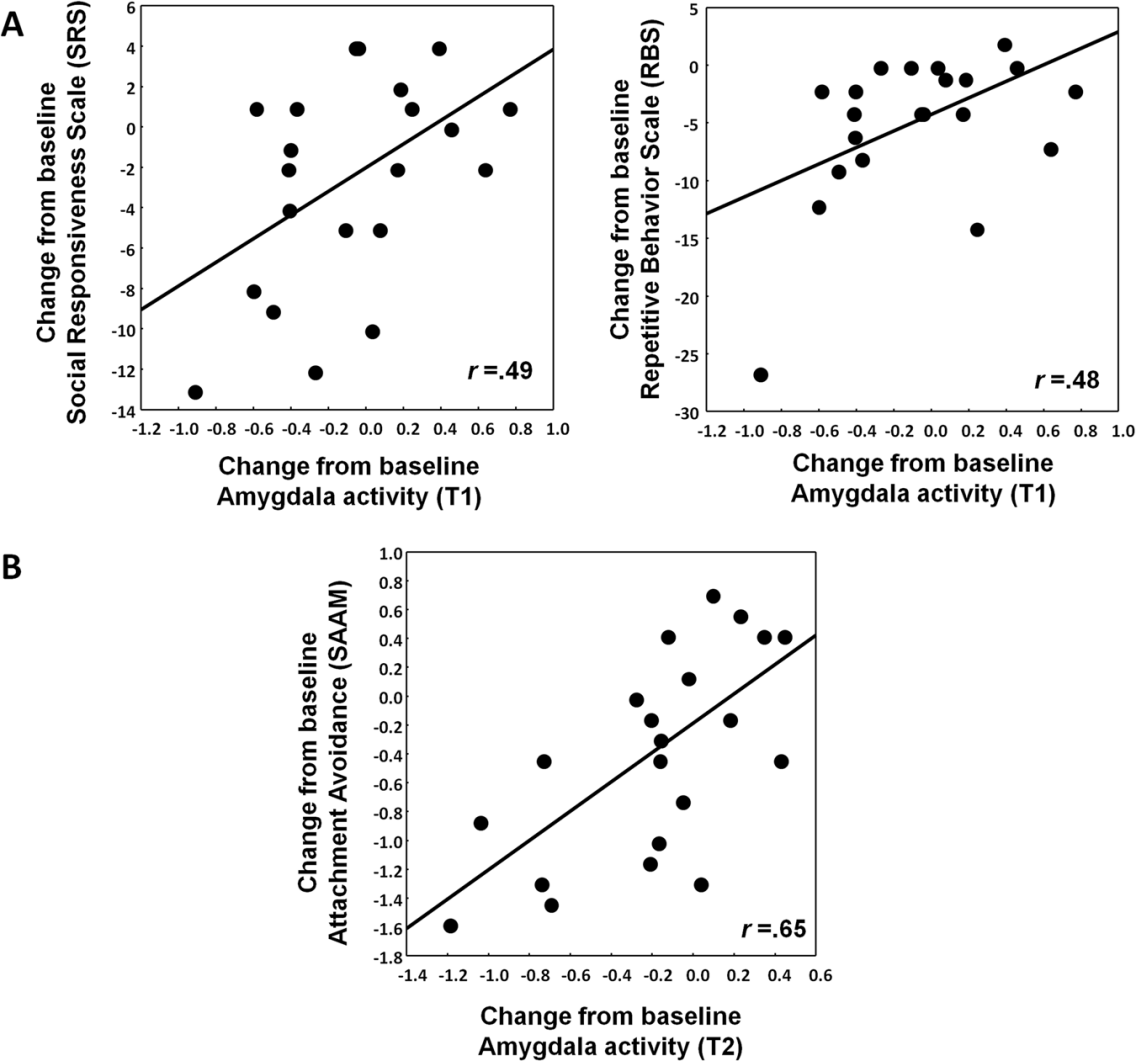
Association between neural changes in amygdala activity and behavioral improvements. Panel **A** visualizes the relationship between oxytocin-induced reductions in amygdala activity immediately after the 4-week multiple-dose treatment (assessment session T1) and behavioral improvements in self-reported social functioning (Social Responsiveness Scale: SRS) and repetitive behaviors (Repetitive behavior scale: RBS). Panel **B** visualizes the relationship between oxytocin-induced reductions in amygdala activity at the 4-week follow-up session (assessment session T2) and behavioral improvements in self-reported attachment avoidance (State Adult Attachment Measure: SAAM). Overall, participants of the oxytocin group with the greatest symptom improvements (negative scores indicate improvement) showed the strongest attenuation of amygdala activity.

Treatment-induced reductions in amygdala activity observed immediately after the multiple-dose treatment (at session T1) were associated with self-reported improvements in social functioning (assessed with the SRS) (*r*_(21)_ = 0.49; *p* = 0.024) and self-reported improvements in repetitive behaviors (assessed with the RBS) (*r*_(21)_ = 0.48; *p* = 0.027) (Fig. 4A). Note however that the relationship with the RBS was in part driven by an outlier-data point, since the effect was rendered tentative using a more outlier-robust correlation test (Spearman rank order *R*_(21)_ = 0.41; *p* = 0.062) and was no longer significant after removal of the outlier-data point (*R*_(20)_ = 0.32; *p* = 0.17).

Further, treatment-induced reductions in amygdala activity observed at the 4-week follow-up session (T2) were significantly associated with self-reported improvements in feelings of avoidant attachment (assessed with the SAAM) (*r*_(21)_ = 0.62; *p* = 0.001) (Fig. 4B). At the one-year follow-up session (T3), no significant brain–behavior associations were identified between neural changes (amygdala or right pSTS activity) and symptom improvements (all, *p* > 0.15). Also no significant associations between neural and behavioral changes were evident in the PL group (all, *p* > 0.13).

## Discussion

In the current study, we investigated the effects of single-dose and multiple-dose IN-OT treatment on neural activation during the processing of emotional states from point-light biological motion in adult men with ASD. Overall, the multiple-dose treatment induced an attenuation in bilateral amygdala activity during the emotion processing task, and notably, these reductions outlasted the intervention until 4 weeks and even one year post-treatment. In terms of neural activity at the level of the pSTS, we mainly identified an acute effect of IN-OT (30 min after single-dose administration), indicating an increase in (bilateral) pSTS activity in the IN-OT, compared to the PL group. However, also at the one-year follow-up session, sustained higher levels of task-related activity within (right) pSTS were observed. Together, these findings indicate that a 4-week IN-OT treatment can induce long-lasting neural changes in core social brain regions.

The observation of increased activation in pSTS regions after single-dose IN-OT administration is consistent with a prior study showing enhanced pSTS recruitment during biological motion processing following IN-OT administration in children with ASD^23^. Similarly, Gordon et al. (2013) showed that single-dose IN-OT administration specifically increased activity in left pSTS during social judgments, but not during nonsocial judgments^24^. Recent meta-analyses of single-dose administration studies including healthy and patient populations consistently identified pSTS regions (middle and superior temporal gyrus) as robust areas of activation after IN-OT administration^22,32,51^. In the current study, we extend findings from these single-dose administration studies by showing that (right) pSTS activation is also altered after multiple-dose treatment with IN-OT. While pSTS activation was generally attenuated in both treatment groups upon repeated presentation of the PLD emotion stimuli, the IN-OT treatment tended to reduce this attenuation/habituation, particularly at the follow-up session, one year post-treatment.

Considering its implicated role in social perception, emotion processing and theory of mind, dysfunction of pSTS regions (particularly right pSTS) has been highlighted as an important neural substrate for the socio-emotional difficulties characteristic of ASD (reviewed in ref. ^52^). For example, compared to neurotypical controls, individuals with ASD have been shown to display reduced pSTS activity upon biological motion processing tasks in previous studies from our lab^59,60^ and other groups^65–69^. The current observation of OT-related effects in pSTS highlights the potential of IN-OT treatment for alleviating dysfunctions in this core social brain area. Overall, the obtained pattern of results for the pSTS region is largely in line with the proposed role of OT in modulating social salience, by facilitating attention to and perception of social signals^18^. The observations are also supportive of the Social adaptation model by Ma et al. (2016), suggesting that especially in individuals that already display low social sensitivity at baseline (such as individuals with ASD), IN-OT may primarily act by enhancing social salience and the neural substrates underlying social processing^20^. Note however that in the current study, no significant relationship was observed between the identified neural changes in pSTS activity and changes in task performance on the point-light emotion recognition task. Moreover, and contrary to our expectations, the placebo group was shown to display a larger performance improvement in emotion task performance, compared to the IN-OT group, albeit only after the single-dose administration, not after the multiple-dose treatment. This finding is at odds with several previous IN-OT administration studies in which improved biological motion perception was demonstrated after a single dose of IN-OT^29–31^. In particular, using a similar task, prior work from our group showed that in neurotypical adult men, a single-dose IN-OT administration improved the recognition of emotional states from PLD when different sets of PLD stimuli were presented pre- and post-treatment^31^. In the current study, the same set of PLD stimuli was presented at each assessment session (albeit in a randomized order), potentially causing general learning effects to interfere with treatment-related effects. Nonetheless, future research is needed to further explore these differential findings and to determine the significance of differential acute versus chronic effects of IN-OT administration on processing of emotional states from point-light biological motion.

In terms of amygdala activity, our study revealed that the IN-OT treatment significantly decreased bilateral amygdala activity (compared to PL) until 4 weeks and even one year post-treatment. Furthermore, exploratory analysis showed that the extent of amygdala attenuation was associated with (long-term) behavioral improvements, particularly in terms of self-reports of social functioning and feelings of attachment avoidance, as well as in terms of repetitive behaviors (although note an outlier data point in this analysis). In previous analyses within the same sample of individuals with ASD, we demonstrated that the 4-week IN-OT treatment significantly altered intrinsic (resting-state fMRI) functional connectivity of the amygdala to core regions of the ‘social brain’ (e.g. orbitofrontal cortex and superior temporal sulcus), indicating that spontaneous activity fluctuations in these brain regions became increasingly de-coupled from the neuronal fluctuations measured in amygdalar regions^53^. Together, these results consistently point toward an overall reduction in amygdala activity and amygdala-related correlated brain activity after multiple-dose IN-OT treatment. We generally anticipate these attenuating effects on amygdala activity to reflect OT’s anxiolytic role in downregulating negative affect, social withdrawal and distress, a notion that is—at least in part—supported by the identified relationships between attenuated amygdala activity and treatment-induced improvements in autism symptoms, including social responsiveness and repetitive behaviors. While the exact link between expressions of repetitive behaviors and difficulties in the social domain is unclear, it has been suggested that at least in a subset of individuals with ASD, the experience of the external (social) milieu as ‘over-arousing’ or even ‘threatening’ may result in an increased ‘need for sameness’ (repetitive behaviors) in order to sustain a level of control over the external surroundings^70,71^. The current results suggest that multiple-dose IN-OT treatment may relieve ASD individuals from this increased ‘over-arousal’ and hence ‘need for sameness’ and provide important indications that these behavioral effects are related to IN-OT-induced reductions in amygdala reactivity. Specifically, it is hypothesized that a recursive halting of excessive amygdala reactivity from the repeated administrations of IN-OT may have relieved individuals from their over-arousal, thereby allowing an increased propensity to engage with the external (social) milieu. In turn, it can be anticipated that the resulting enhancement of ‘positive’ (social) experiences may have further contributed to an adaptive and recursive re-shaping of amygdala functioning in an experience-dependent manner (hence the observation of long-lasting neural and behavioral changes up to one year post-treatment).

In the current study, effect sizes of the multiple-dose treatment were qualitatively different compared to the identified single-dose effect sizes. In terms of pSTS activity, the effect was most pronounced after single-dose administration, whereas in terms of amygdala attenuation, the effect was most pronounced after the multiple-dose treatment. These observations provide indications that the neural effects after single-dose compared to multiple-dose IN-OT treatment are different, and importantly, that the differentially induced single-dose versus multiple-dose effects may be region-specific or network-specific. In a prior multiple-dose administration study, Watanabe et al. (2015) investigated the neural effects of a 6-week IN-OT treatment on anterior cingulate and prefrontal cortex activity during a social judgment task, but in this study, effect sizes of the multiple-dose treatment were found to be qualitatively similar to effect sizes of a previous single-dose administration study^11^. However, MRI scanning for assessing the 6-week treatment effect was performed only 15 or 40 min after the last nasal spray administration. Since this period corresponds to the optimal time frame for assessing acute, single-dose effects of IN-OT administration, the possibility cannot be ruled out that the reported multiple-dose effect—at least in part—reflected an acute effect of exogenously administered OT. In contrast, in the current study, MRI scanning for assessing the multiple-dose effect (T1) was performed at least 24 h after the last IN-OT administration, rendering the observed neural effects at session T1 less susceptible to reflect an acute effect of IN-OT administration. Also the neural changes at the 4-week and one-year follow-up sessions are interpreted to solely reflect long-lasting adaptations in neural functioning due to OT’s recursive action on these circuits.

The study has some limitations worth noting. In accordance with most prior studies assessing single-dose effects of IN-OT, the current study adopted a fixed dose of 24 IU. It cannot be ascertained however that a similar pattern of results would have emerged at a different dosing. For example, a prior study investigating the effect of IN-OT on fear processing in the amygdala showed that a dose of 24 IU significantly attenuated amygdala reactivity, while opposite effects were induced after a higher dose of 48-IU dose^72^. Also differential effects of lower doses have been demonstrated, indicating that a low dose of 8 IU administered using a breath powered delivery system was more efficacious for modulating overt social salience, compared to a dose of 24 IU^73^. Further, while the here adopted daily dose of 24 IU (administered in the morning) is in accordance with a prior study assessing multiple-dose effects in ASD^16^, it should be noted that the majority of prior multiple-dose OT studies administered two doses/day (one in the morning and one in the afternoon, e.g. Watanabe et al. 2015)^11^. Together, these studies highlight that more work is needed to delineate the most optimal dosing schema, including the most optimal timing and administration lengths for multiple dose administration designs. Further, since this is the first multiple-dose administration study directly investigating changes in amygdala functioning following IN-OT treatment, future studies may be warranted to explore whether the observed long-term attenuating effects up to one year post-treatment are specific to (male) adults with ASD, or whether they will generalize across gender, other (patient) populations and developmental stages. Indeed, meta-analyses of single-dose administration studies, have highlighted gender and psychopathology as important factors for moderating the directionality of single-dose IN-OT’s effects on brain function, indicating, for example, more consistent amygdala attenuation in men compared to women^20,22^. Other factors that have been highlighted to modulate (the direction of) treatment responses relate to contextual factors, such as stimulus valence or the nature of the presented task (reviewed in refs. ^32,51^). In the current study, neural responses were measured upon processing point-light stimuli conveying either a negative (anger) of positive (happiness) emotional state, and while similar treatment effects were evident irrespective of valence, it cannot be ascertained that these effects would generalize across different types of emotional states, for example fear or disgust, or for neutral (social) stimuli expressing no particular emotional evocativeness. Future studies are therefore warranted to explore whether the reported OT-induced changes in task-related brain activity will generalize across different stimulus types or in different task contexts. Finally, it should be noted that placebo in this study was normal saline water. Although participants were not reliably able to detect whether they received the active compound (the proportion of participants that believed they had received the IN-OT treatment was not significantly larger in the actual OT group (28.57%) compared to the PL group (11.11%) (*p* = 0.18)); future studies are recommended to use placebo with identical inactive ingredients as used in the intranasal syntocinon administrations.

To conclude, our study revealed that multiple-dose IN-OT treatment is effective for inducing long-lasting adaptations in core social brain regions (pSTS and amygdala) that outlast the 4-week period of actual IN-OT administration until 4 weeks and even one year post-treatment. These observations hold important clinical implications for ASD and other neuropsychiatric conditions for which IN-OT is considered as a potential treatment.

## Supplementary information

Supplementary Materials

## Data Availability

Data set discussed in this manuscript can be made available (in anonymized format) upon reasonable request from qualified investigators.
