# Supplementary material for: Oxytocin treatment attenuates amygdala activity in autism: a treatment-mechanism study with long-term follow-up
Source: Transl Psychiatry. 2020 Nov 6;10:383. doi: 10.1038/s41398-020-01069-w (PMC7648620; doi:10.1038/s41398-020-01069-w)
Supplement: Supplementary file 1 — Supplementary Materials [file 41398_2020_1069_MOESM1_ESM.pdf]

Supplementary Materials

Oxytocin treatment attenuates amygdala activity in autism: a treatment-mechanism study with long-term follow-up

Sylvie Bernaerts, Bart Boets, Jean Steyaert, Nicole Wenderoth, Kaat Alaerts

Supplementary methods

1.1. Participants - included sample size

Several studies explored the effect of single-dose IN-OT administration on changes in task-related fMRI brain activity in patients with ASD (8–12). In one previous randomized, placebo-controlled trial, Watanabe et al. (13) adopted a crossover design to assess the effect of multiple-dose IN-OT treatment (six weeks of daily doses) on task-related fMRI activity during a social judgement task in 20 patients with ASD. Significant effects (large-size) were reported for 17 patients who completed the OT/PL crossover treatment. Considering this previous crossover study assessing the effects of multiple-dose IN-OT treatment on task-related fMRI activity in patients with ASD and the lack of prior studies using parallel designs, the current sample size was set at a comparable sample size.

1.2. Side effect screening.

After each week of the four-week treatment, participants were screened for potential adverse events, side effects or changes in mood. Detailed information on the reporting of side-effects and changes in mood is provided in Bernaerts et al. (2020) (1). In short, only minimal, non-treatment specific side effects were reported. Finally, at the end of the trial, participants were asked if they thought they had received OT or PL. The majority of participants thought they had received the PL treatment (77.5%). The proportion of participants that believed they had received the OT-treatment was not significantly larger in the actual OT-group (28.5%), compared to the PL-group (17.6%) (p= .46).

1.3. MRI data acquisition and handling

MRI data acquisition. A 3.0 Tesla Philips Achieva Ds MR-scanner with a 32-channel phased-array head-coil was used to acquire anatomical images and the two task-related fMRI runs.
Note that since participants were recruited to participate in a larger study assessing the (neural and behavioral) effects of multiple-dose treatment with IN-OT, the fMRI scanning protocol additionally included two other scan modalities (not part of the current report): (i) resting-state fMRI scanning (acquired prior to the task-based fMRI runs) (14) and (ii) diffusion tensor imaging (acquired after the task-based fMRI runs).

MRI scanning parameters. Anatomical imaging consisted of a high resolution structural volume acquired using a coronal three-dimensional turbo field echo T1-weighted sequence with the following parameters: 182 contiguous coronal slices covering the whole brain and brainstem, slice thickness = 1.2 mm; repetition time (TR) = 9.4 ms; echo time (TE) = 3.0 ms; matrix size = 256 × 256; field-of-view (FOV) = 250 × 250 x 218.40 mm^3; acquisition time = 1 min 43s. For the two task-related fMRI scans a T2* weighted gradient echo - echo planar imaging (GE-EPI) sequence was used with the following parameters: TR = 3000 ms; TE = 30 ms; matrix size = 96 × 96; FOV = 210 x 210 x 140.20 mm^3; flip angle 90°; slice thickness = 2.5 mm, 0.2 mm gap; axial slices = 52; 127 functional volumes; total acquisition time = 6 min 39s.

Preprocessing. All functional images were corrected for differences in slice acquisition time by temporal interpolation to the middle slice (reference=26), realigned to the reference (mean) image and

co-registered to each subject's T1 anatomical image. Images were then normalized to the standard EPI template of the Montreal Neurological Institute (MNI) space using the segmented anatomical image, resampled into 2 mm isotopic voxels and smoothed with an 8-mm full width at half maximum Gaussian kernel. A high-pass filter with a cutoff of 256s was used. Mean framewise displacement (FD) was calculated for each participant to assess potential differences in in-scanner head movement between groups across sessions. As shown in **Supplementary Figure 2,** no significant differences were evident in mean FD between groups across assessment sessions.

Supplementary Table 1.

Detailed information on comorbidities and medication use for participants of the oxytocin and placebo treatment groups.

Comorbidities were screened through self-report (with the explicit mentioning of examples in the screening interview including e.g., ADHD, depression, dyscalculia, dyslexia). Current psychoactive medication use was defined as use within three months before study enrollment. None of the participants reported a change in medication regime between the baseline (T0) and four-week follow-up session (T2). Two participants reported a change in medication use at the one-year follow-up session (T3).

	**Comorbidities**	**Medication use (T0 till T2)**	**Change Medication use at T3**
**Oxytocin group**	**N= 7**	**N= 5**	**N=2**
**i**	ADHD	Abilify, Tegretol	
**ii**	Depression	Welbutrine XR, Leviron, Cymbalta	Cymbalta, Trazolan, Edronax, Depakine
**iii**	Depression, ADD	Trazodone Mylan, Medikinet	
**iv**	Bipolar disorder	Maniprex, Bellozal, Mometasone	
**v**	ADHD,Dyslexia	/	
**vi**	ADHD, Depression	/	
**vii**	Dyslexia	/	
**viii**	/	Risperdal, Venlafaxine	
**ix**	/	/	Sertraline
**Placebo group**	**N= 2**	**N= 2**	
**i**	ADHD	/	
**ii**	ADHD	/	
**iii**	/	Zolpidem, Remergon, Rilatine	
**iv**	/	Trazodone, Escitalopram	

ADHD: attention deficit hyperactivity disorder; ADD: attention deficit disorder

Supplementary Table 2

Treatment-induced changes in behavior.

As reported in more detail in Bernaerts et al. (2020) (1), we previously explored behavioral improvements as a result of the four-week IN-OT treatment in the same patient sample in terms of social functioning (Social Responsiveness Scale - Adult version: SRS-A), repetitive behaviors (Repetitive Behavior Scale – Revised: RBS-R) and attachment avoidance (State Adult Attachment Measure: SAAM).

- The SRS-A (self-report) (64 items) (2) comprises four subscales examining social communication, social awareness, social motivation and rigidity/repetitiveness, using a four-point Likert-scale. SRS-A raw total scores were adopted.
- The RBS-R (self-report) (43 items) (3) examines a heterogeneous set of repetitive behaviors including stereotypic behavior, self-injurious behavior, compulsive behavior, ritualistic behavior, sameness behavior and restricted interests behavior, using a four-point Likert-scale.
- The SAAM (self-report) (4) comprises three subscales, of which one subscale assesses attachment avoidance (e.g., "If someone tried to get close to me, I would try to keep my distance") (7 items) using a seven-point Likert-scale.

In short, behavioral improvements were evident immediately after treatment (T1) and until four weeks (T2) and one year (T3) post-treatment in repetitive behaviors (RBS-R) and feelings of avoidant attachment (SAAM). While the oxytocin group also reported improvements in social symptoms (SRS-A), these improvements were not treatment-specific (i.e., comparable improvements were evident in the placebo group).

The table below lists for each questionnaire the mean pre-to-post change scores separately for each treatment group (oxytocin, placebo) and assessment session (T1, T2, T3). *T*- and *p*-values correspond to single-sample *t*-tests assessing within-group changes from baseline separately for the oxytocin and placebo group. Cohen's *d* effect sizes of between-group differences (change from baseline_{OT}–change from baseline_{PL})/pooled SD) are reported where 0.2 is indicative of a small effect, 0.5 a medium effect and 0.8 a large effect.

	Oxytocin				Placebo				Between-group difference
	N	Mean ± SD	*T*-value	*p*	N	Mean ± SD	*T*-value	*p*	Cohen's *d*
**Multiple-dose effect (T1)**									
SRS-A	22	-5.55 ± 11.40	-2.28	0.033	18	-1.06 ± 10.01	-0.45	0.66	-0.42
RBS-R	22	-4.77 ± 6.47	-3.46	0.002	17	-1.76 ± 4.75	-1.53	0.15	**-0.63**
SAAM avoidance	22	-0.40 ± 0.71	-2.63	0.016	18	0.06 ± 0.98	0.24	0.81	**-0.61**
**Four-week follow-up (T2)**									
SRS-A	22	-5.64 ± 12.57	-2.10	0.048	18	-7.67 ± 12.09	-2.69	0.015	0.22
RBS-R	22	-4.91 ± 6.33	-3.64	0.002	17	-2.35 ± 3.43	-2.83	0.012	**-0.50**
SAAM avoidance	22	-0.38 ± 0.70	-2.58	0.018	18	-0.06 ± 0.76	-0.35	0.73	**-0.53**
**One-year follow-up (T3)**									
SRS-A	22	-8.59 ± 20.95	-1.92	0.07	18	-6.72 ± 21.01	-1.36	0.19	-0.12
RBS-R	22	-4.91 ± 9.46	-2.43	0.02	17	-0.41 ± 4.27	-0.40	0.70	**-0.98**
SAAM avoidance	22	-0.52 ± 1.18	-2.07	0.05	18	0.0 ± 0.75	0.00	1.00	**-0.80**

SRS-A = Social Responsiveness Scale adult version, RBS-R = Repetitive Behavior Scale – Revised, SAAM = State Adult Attachment Measure, Negative scores indicate pre-to-post improvement.

T- and *p*-values correspond to single-sample *t*-tests assessing within-group changes from baseline separately for the oxytocin and placebo group. Cohen's *d* effect sizes of between-group differences (change from baseline_{OT}–change from baseline_{PL})/pooled SD) are reported where 0.2 is indicative of a small effect, 0.5 a medium effect and 0.8 a large effect. Data printed in bold show Cohen's *d* effect sizes equal to or larger than .50 (medium-sized effect).

Supplementary Figure 1.

Visualization of experimental design. (**A**) Participants completed two runs, each consisting of three blocks of the emotion recognition task, interleaved with three color task blocks (48s/block). All task blocks were separated by fixation blocks (12s rest period), during which participants fixated on a white cross. All trials lasted 4s, such that stimulus presentation was jittered with respect to image acquisition (TR = 3s). Instructions were provided verbally at the start of the test and on the monitor at the start of each test block (4s). (**B**) Point-light displays (PLDs) consisted of twelve moving white dots against a black background, representing the motion of the main joints of the human body (ankles, knees, hips, wrists, elbows and shoulders). Response options were displayed at the bottom of the screen, which corresponded to response buttons of the response box that the participants used while lying in the scanner.

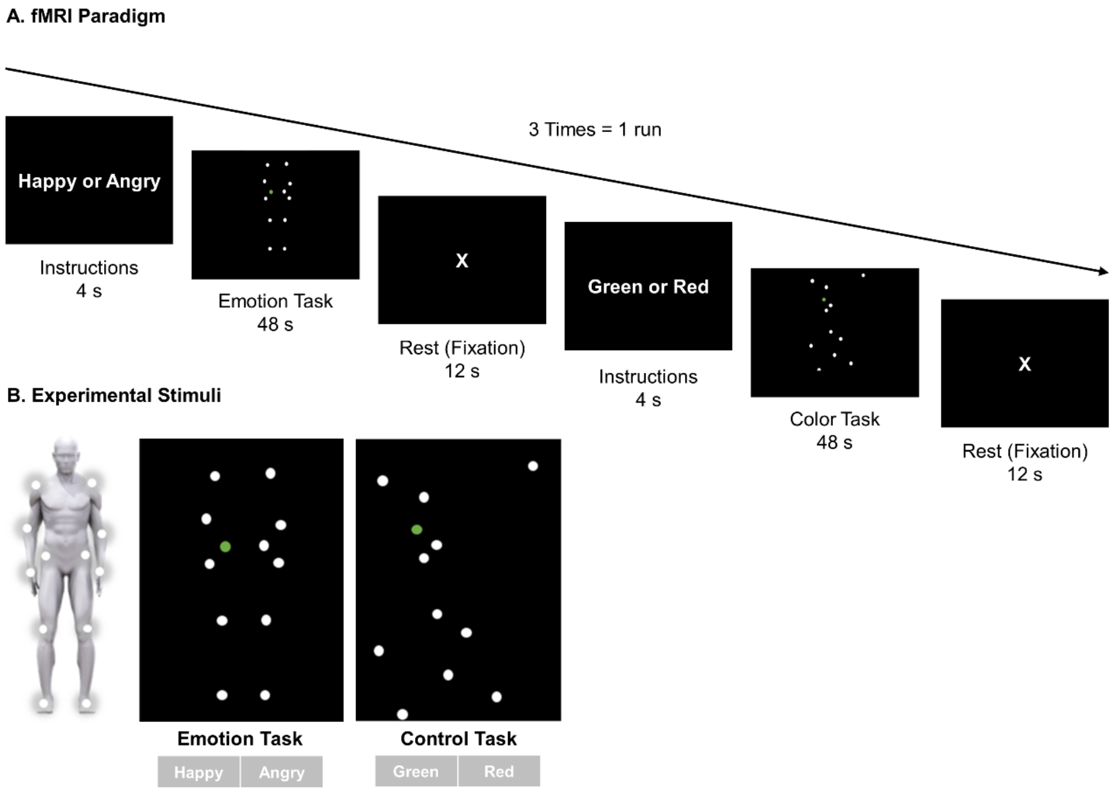

Supplementary Figure 2.

Head motion analysis of the task-based fMRI scans.

Mean frame-wise displacement (FD) (in mm) was calculated for each participant to assess potential differences in in-scanner head movement between treatment groups and assessment sessions. A mixed-effects analyses with 'Subject' as random factor and the factors 'Treatment' (OT, PL) and 'Session' (T0, SD, T1, T2, T3) as fixed factor revealed no main effects of treatment ($F(1,138)=.001$, $p=.97$) or session ($F(4,138)=2.06$, $p=.09$), nor a treatment-by-session interaction ($F(4,138)=.12$, $p=.97$), indicating no significant differences in mean FD between groups across test sessions.

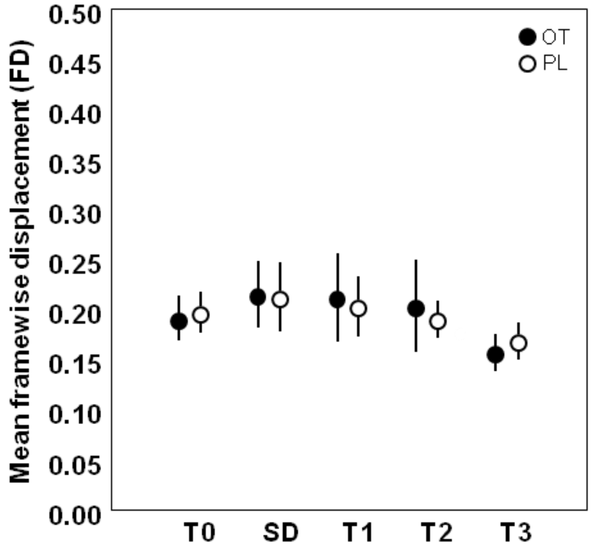

Supplementary Figure 3.

Visualization of brain regions showing brain activity during the emotion task at baseline.

A whole-brain one-sample t-test analysis was performed to identify regions with brain activity during the emotion task (> control task) at the baseline session (T0) (across groups) ($p < .05$, family-wise error corrected for multiple comparisons) (red-orange grading). As visualized, the adopted regions of interest (ROI) in bilateral posterior superior temporal sulcus (pSTS) (10-mm-radius spheres with MNI-coordinates [left: -55, -52, 12] [right: 55, -52, 10]) and bilateral amygdala (FSL Harvard-Oxford subcortical atlas) (visualized in blue) showed reliable brain activity during the emotion task (overlap visualized in purple).

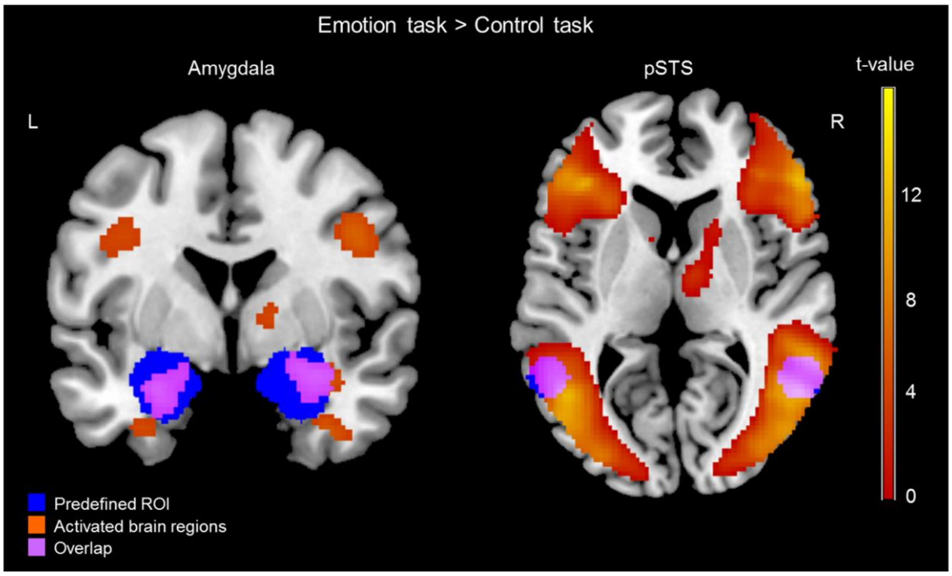

Hemisphere	Anatomical label	Peak MNI coordinates			Cluster size	T-value
		X	Y	Z		
R	Inferior temporal gyrus	46	-70	-8	7214	14.59
L	Fusiform gyrus	-42	-42	-20	4757	15.39
R	Inferior frontal gyrus - including insula	52	34	14	4189	13.16
L	Inferior frontal gyrus - including insula	-46	28	18	3440	12.04
L	Prefrontal gyrus - supplementary motor area	-2	24	50	850	10.99
L	Inferior parietal gyrus - intraparietal sulcus	-30	-54	42	522	7.35
R	Cerebellum	14	-76	-34	342	9.03
R	Amygdala	28	-4	-16	72	6.64
L	Amygdala	-26	-4	-20	45	7.11
L	Inferior parietal gyrus – supramarginal gyrus	-50	-36	24	37	6.30
R	Inferior temporal gyrus - fusiform face area	38	-4	-40	14	7.26
L	Medial temporal gyrus	-30	-4	-38	17	7.06

Whole brain one-sample t-test analysis: $p<.05$, familywise error-corrected; Extent threshold: $k=10$ voxels. MNI= Montreal Neurological Institute. L= Left. R= Right.

References

1. Bernaerts S, Boets B, Bosmans G, Steyaert J, Alaerts K (2020): Behavioral effects of multiple-dose oxytocin treatment in autism: a randomized, placebo-controlled trial with long-term follow-up. *Mol Autism* 11: 6.

2. Constantino JN, Gruber CP (2005): *Social Responsiveness Scale*. Los Angeles, CA: Western Psychological Services.

3. Lam KSL, Aman MG (2007): The repetitive behavior scale-revised: Independent validation in individuals with autism spectrum disorders. *J Autism Dev Disord* 37: 855–866.

4. Gillath O, Hart J, Noftle EE, Stockdale GD (2009): Development and validation of a state adult attachment measure (SAAM). *J Res Pers* 43: 362–373.

5. American Psychiatric Association (2013): *Diagnostic and Statistical Manual of Mental Disorders (DSM-5®)*, 5th ed. ((V. A. Arlington, editor)). American Psychiatric Association. Retrieved February 4, 2016, from https://books.google.com/books?hl=nl&lr=&id=-JivBAAAQBAJ&pgis=1

6. Lord C, DiLavore P, Gotham K, Guthrie W, Luyster R, Risi S, Rutter M (2012): *Autism Diagnostic Observation Schedule: ADOS-2*, 2nd ed. Los Angeles: Western Psychological Services. Retrieved June 19, 2019, from https://www.worldcat.org/title/autism-diagnostic-observation-schedule-ados-2/oclc/851410387

7. Wechsler D, Coalson D, Raiford S (2008): *WAIS IV Technical and Interpretive Manual*. San Antonio: NCS Pearson.

8. Aoki Y, Yahata N, Watanabe T, Takano Y, Kawakubo Y, Kuwabara H, *et al.* (2014): Oxytocin improves behavioural and neural deficits in inferring others' social emotions in autism. *Brain* 137: 3073–86.

9. Gordon I, Jack A, Pretzsch CM, Vander Wyk B, Leckman JF, Feldman R, Pelphrey KA (2016): Intranasal Oxytocin Enhances Connectivity in the Neural Circuitry Supporting Social Motivation and Social Perception in Children with Autism. *Sci Rep* 6: 35054.

10. Gordon I, Vander Wyk BC, Bennett RH, Cordeaux C, Lucas M V., Eilbott JA, *et al.* (2013): Oxytocin enhances brain function in children with autism. *Proc Natl Acad Sci* 110: 20953–20958.

11. Domes G, Heinrichs M, Kumbier E, Grossmann A, Hauenstein K, Herpertz SC (2013): Effects of Intranasal Oxytocin on the Neural Basis of Face Processing in Autism Spectrum Disorder. *Biol Psychiatry* 74: 164–171.

12. Watanabe T, Abe O, Kuwabara H, Yahata N, Takano Y, Iwashiro N, *et al.* (2014): Mitigation of sociocommunicational deficits of autism through oxytocin-induced recovery of medial prefrontal activity: a randomized trial. *JAMA psychiatry* 71: 166–75.

13. Watanabe T, Kuroda M, Kuwabara H, Aoki Y, Iwashiro N, Tatsunobu N, *et al.* (2015): Clinical and neural effects of six-week administration of oxytocin on core symptoms of autism. *Brain* 138: 3400–12.

14. Alaerts, K., Bernaerts, S., Steyaert, J., and Wenderoth, N. (2020) Oxytocin treatment induces long-lasting adaptations within amygdala circuitry in autism. *Neuropyschopharmacology* 45, 1141-1149.

CONSORT 2010 checklist of information to include when reporting a randomised trial*

Section/Topic	Item No	Checklist item	Reported on page No
**Title and abstract**			
	1a	Identification as a randomised trial in the title	NA
	1b	Structured summary of trial design, methods, results, and conclusions (for specific guidance see CONSORT for abstracts)	3
**Introduction**			
Background and objectives	2a	Scientific background and explanation of rationale	4-5
	2b	Specific objectives or hypotheses	5
**Methods**			
Trial design	3a	Description of trial design (such as parallel, factorial) including allocation ratio	6
	3b	Important changes to methods after trial commencement (such as eligibility criteria), with reasons	NA
Participants	4a	Eligibility criteria for participants	7
	4b	Settings and locations where the data were collected	6
Interventions	5	The interventions for each group with sufficient details to allow replication, including how and when they were actually administered	7 - 8
Outcomes	6a	Completely defined pre-specified primary and secondary outcome measures, including how and when they were assessed	7-8 + Suppl. p 2-3
	6b	Any changes to trial outcomes after the trial commenced, with reasons	NA
Sample size	7a	How sample size was determined	7 + Suppl. p 2
	7b	When applicable, explanation of any interim analyses and stopping guidelines	NA

Randomisation:			
Sequence generation	8a	Method used to generate the random allocation sequence	7
	8b	Type of randomisation; details of any restriction (such as blocking and block size)	-
Allocation concealment mechanism	9	Mechanism used to implement the random allocation sequence (such as sequentially numbered containers), describing any steps taken to conceal the sequence until interventions were assigned	-
Implementation	10	Who generated the random allocation sequence, who enrolled participants, and who assigned participants to interventions	7
Blinding	11a	If done, who was blinded after assignment to interventions (for example, participants, care providers, those assessing outcomes) and how	7
	11b	If relevant, description of the similarity of interventions	NA
Statistical methods	12a	Statistical methods used to compare groups for primary and secondary outcomes	8-10
	12b	Methods for additional analyses, such as subgroup analyses and adjusted analyses	8-10 + 12
**Results**			
Participant flow (a diagram is strongly recommended)	13a	For each group, the numbers of participants who were randomly assigned, received intended treatment, and were analysed for the primary outcome	Fig. 1. CONSORT Flow diagram
	13b	For each group, losses and exclusions after randomisation, together with reasons	Fig. 1. CONSORT Flow diagram
Recruitment	14a	Dates defining the periods of recruitment and follow-up	Fig. 1. CONSORT Flow diagram
	14b	Why the trial ended or was stopped	-

Baseline data	15	A table showing baseline demographic and clinical characteristics for each group	Table 1 + Suppl. Table 1
Numbers analysed	16	For each group, number of participants (denominator) included in each analysis and whether the analysis was by original assigned groups	Fig. 1. CONSORT Flow diagram
Outcomes and estimation	17a	For each primary and secondary outcome, results for each group, and the estimated effect size and its precision (such as 95% confidence interval)	11-12
	17b	For binary outcomes, presentation of both absolute and relative effect sizes is recommended	NA
Ancillary analyses	18	Results of any other analyses performed, including subgroup analyses and adjusted analyses, distinguishing pre-specified from exploratory	12
Harms	19	All important harms or unintended effects in each group (for specific guidance see CONSORT for harms)	NA
**Discussion**			
Limitations	20	Trial limitations, addressing sources of potential bias, imprecision, and, if relevant, multiplicity of analyses	15-16
Generalisability	21	Generalisability (external validity, applicability) of the trial findings	15-16
Interpretation	22	Interpretation consistent with results, balancing benefits and harms, and considering other relevant evidence	13-16
**Other information**			
Registration	23	Registration number and name of trial registry	7
Protocol	24	Where the full trial protocol can be accessed, if available	7
Funding	25	Sources of funding and other support (such as supply of drugs), role of funders	17

*We strongly recommend reading this statement in conjunction with the CONSORT 2010 Explanation and Elaboration for important clarifications on all the items. If relevant, we also recommend reading CONSORT extensions for cluster randomised trials, non-inferiority and equivalence trials, non-pharmacological treatments, herbal interventions, and pragmatic trials. Additional extensions are forthcoming: for those and for up to date references relevant to this checklist, see www.consort-statement.org.
